# SGLT2 Inhibitor—Dapagliflozin Attenuates Diabetes-Induced Renal Injury by Regulating Inflammation through a CYP4A/20-HETE Signaling Mechanism

**DOI:** 10.3390/pharmaceutics15030965

**Published:** 2023-03-16

**Authors:** Batoul Dia, Sahar Alkhansa, Rachel Njeim, Sarah Al Moussawi, Theresa Farhat, Antony Haddad, Mansour E. Riachi, Rashad Nawfal, William S. Azar, Assaad A. Eid

**Affiliations:** 1 Department of Anatomy, Cell Biology and Physiological Sciences, Faculty of Medicine, American University of Beirut, Riad El Solh, Beirut 1107-2020, Lebanon; 2 AUB Diabetes, Faculty of Medicine and Medical Center, American University of Beirut, Riad El Solh, Beirut 1107-2020, Lebanon

**Keywords:** diabetic kidney disease, SGLT2 inhibitor, 20-HETE, oxidative stress, inflammatory markers

## Abstract

Diabetic kidney disease (DKD) is a serious complication of diabetes, affecting millions of people worldwide. Inflammation and oxidative stress are key contributors to the development and progression of DKD, making them potential targets for therapeutic interventions. Sodium-glucose cotransporter 2 inhibitors (SGLT2i) have emerged as a promising class of drugs, with evidence demonstrating that they can improve renal outcomes in people with diabetes. However, the exact mechanism by which SGLT2i exert their renoprotective effects is not yet fully understood. This study demonstrates that dapagliflozin treatment attenuates renal injury observed in type 2 diabetic mice. This is evidenced by the reduction in renal hypertrophy and proteinuria. Furthermore, dapagliflozin decreases tubulointerstitial fibrosis and glomerulosclerosis by mitigating the generation of reactive oxygen species and inflammation, which are activated through the production of CYP4A-induced 20-HETE. Our findings provide insights onto a novel mechanistic pathway by which SGLT2i exerts their renoprotective effects. Overall, and to our knowledge, the study provides critical insights into the pathophysiology of DKD and represents an important step towards improving outcomes for people with this devastating condition.

## 1. Introduction

Diabetic kidney disease (DKD) is a debilitating complication and a major contributor to all-cause mortality in patients with diabetes. Several risk factors contribute to the development of DKD, including poor glycemic control, hypertension, smoking, dyslipidemia, as well as multiple genetic and environmental factors [1]. DKD is characterized by glomerular, vascular, tubular, and interstitial damage that initially develops in the absence of clinically measurable dysfunction. The current conventional therapies to prevent DKD and slow its progression include intensive blood glucose control [2,3,4,5], blood pressure regulation and renin-angiotensin-aldosterone system (RAAS) blockade [6,7,8]. Despite the importance of these treatment modalities, the risk of developing end-stage renal disease (ESRD) in patients with type 2 diabetes (T2DM) remains considerably high. In that regard, several studies have focused on identifying the signaling pathways indicating the onset of DKD as well as testing new potential biomarkers to achieve the earlier detection of diabetic kidney disease such as NETosis, a novel form of neutrophil-related cell death, and Neutrophil Gelatinase-Associated Lipocalin, to name a few [9,10,11]. Therefore, new therapeutic approaches are needed to reduce the onset of DKD and to curb its progression to ESRD.

Among the new generation of glucose-lowering oral drugs, inhibitors of the sodium-glucose cotransporter 2 (SGLT2) have been identified to have a potential role in lowering the risk of renal complications in patients with diabetes [12,13,14,15,16]. The nephroprotective role of SGLT2 inhibitors (SGLT2i) is not only a consequence of correcting hyperglycemia by significantly decreasing HbA1c levels and reducing body weight [17,18,19], but is also a result of their anti-inflammatory, antifibrotic, and antioxidative stress effects in renal tissues [20,21,22].

Dapagliflozin is one of the SGLT2 inhibitors that has been demonstrated to have cardiac and renoprotective effects in clinical trials such as DECLARE-TIMI 58 [15]. Several reports have attributed the substantial benefits of dapagliflozin on renal function to the attenuation of oxidative stress, apoptosis, ER stress, and inflammation [23,24]. Despite these significant findings, the exact mechanisms by which dapagliflozin exerts its nephroprotective properties are yet to be elucidated.

Arachidonic acid (AA) is metabolized to 20-hydroxyeicosatetraenoic acid (20-HETE) by the cytochrome P450 (CYP) 4A and 4F families of enzymes, with cytochrome P450 (CYP) of the 4A family being the most abundant in mice kidney tissue [25]. Additionally, CYP4A, particularly the CYP4A12a isoform, is the predominant 20-HETE synthase in the mouse kidney [26,27]. The exact contribution of CYP4F isoforms to 20-HETE production in mouse models is not well established. 20-HETE is a powerful vasoconstrictor involved in the regulation of hemodynamics and extracellular fluid volume through tubular and vascular mechanisms [28,29]. Moreover, CYPs are heme-containing monooxygenases. Therefore, the aberrant redox cycling of these enzymes leads to the formation of superoxide(O_2_^−^)/hydrogen peroxide(H_2_O_2_), rendering them a significant source of oxidative stress in different tissues, including renal tissue [30,31,32]. Published data by our group amongst other findings describes the contribution of AA-metabolizing CYP enzymes and their metabolites in inducing reactive oxygen species (ROS) production in the tubules and glomeruli of the kidneys, leading to proteinuria, cellular injury, and apoptosis [29,30,33,34,35,36,37]. CYPs-generated 20-HETE might therefore be involved in the overall decline in the renal function observed in DKD [38].

Amongst the pathophysiological changes caused by diabetes, inflammation has been demonstrated to play a key role contributing to the aberrant metabolism and oxidative stress in DKD [39]. The infiltration of immune cells into the renal tissue and the circulation of proinflammatory molecules have been demonstrated to be increased in both animal models and patients with DKD [40]. Several cytokines and chemokines are thought to play a crucial role in kidney diseases. The chemokine monocyte chemoattractant protein 1 (MCP1) [41] was found to be associated with a decline in the renal function of patients with DKD [42,43,44]. Several other proinflammatory cytokines are believed to contribute to DKD, including the tumor necrosis factor alpha (TNF-α) [45,46,47,48,49], interleukin 6 (IL-6) [50], interleukin 1-beta (IL-1β) [51,52] and interleukin 17 (IL-17) [53].

In this study, we determine the renoprotective effect of dapagliflozin in a high-fat diet (HFD) streptozotocin (STZ)-induced T2DM mouse model. We also highlight, for the first time, the interaction between the SGLT2i and the CYP4A/20-HETE axis that leads to a reduction in ROS production, oxidative stress, and inflammation in the renal cortices of mice. Our findings describe a new mechanistic pathway by which SGLT2i interacts with the CYP4A/20-HETE axis to mediate its renoprotective role in type 2 diabetes.

## 2. Materials and Methods

### 2.1. Animal Models

All animal procedures were conducted in accordance with the American University of Beirut Animal Care and Use Committee guidelines (IACUC protocol number 19-04-523). Ten-week-old male C57BL/6 mice were divided into four groups of four animals each. Type 2 diabetes was induced by maintaining the mice on an HFD containing 60% kcal from fat for four weeks, followed by 3 consecutives intraperitoneal (i.p.) injections of 55 mg/kg body weight of STZ (Sigma-Aldrich, St. Louis, MO, USA) dissolved in citrate buffer (pH 4.5). These mice were maintained on HFD throughout the study until sacrifice. Four weeks after diabetes onset, diabetic mice were divided into 3 different groups: (1) untreated type 2 diabetic group, (2) type 2 diabetic group treated with 1.5 mg/kg dapagliflozin (Farxiga) i.p. daily for 8 weeks and (3) type 2 diabetic group treated with 2 units of insulin (Actrapid) i.p. daily for 8 weeks. Age-matched male C75BL/6 mice maintained on a standard rodent chow (10% of kcal from fat) and injected with 3 consecutive doses of sodium citrate buffer after 4 weeks of the study initiation (0.01 M, pH 4.5) were used as a control group. In parallel experiments, 10-week-old male FVB/NJ mice were rendered type 2 diabetic, as described above. Four weeks after diabetes onset, diabetic mice were divided into the following groups of four animals each: (1) untreated type 2 diabetic group; (2) type 2 diabetic group treated daily with 5 mg/kg of HET0016 administered subcutaneously for 10 weeks. Age-matched male FVB/NJ mice labeled as the control group were maintained on a standard rodent chow containing 10% of kcal from fat and injected with 3 consecutive doses of sodium citrate buffer after 4 weeks of the study initiation (0.01 M, pH 4.5). Animals had ad libitum access to food and water maintained at a temperature-controlled room with 12 h alternating light/dark cycles throughout the whole study period. Body weight and blood glucose were measured weekly. Before sacrifice, mice were placed in metabolic cages for urine collection. Urine albumin to creatinine ratio (UACR) was measured using a mouse albumin enzyme-linked immunosorbent assay (ELISA) quantification kit (Bethyl Laboratories) and expressed as micrograms of albumin/24 h. Animals were sacrificed by exsanguination under anesthesia. Both kidneys were removed and weighed. A slice of kidney cortex at the pole was fixed with 4% formalin for immune-histochemical analysis or flash-frozen in liquid nitrogen and stored at −80 °C for Western blot, PCR, enzymatic assays, microscopy, and image analysis.

### 2.2. Immunohistochemical Analysis

Renal cortical tissues from each group were fixed in a 4% formalin solution and embedded in a paraffin block. Samples were cut into 4-µm-thick sections and placed on glass slides. The kidney sections were then stained with a periodic acid Schiff (PAS) reagent to assess glomerulosclerotic index and mesangial accumulation, as previously described [54], and Masson trichrome (MT) stain to evaluate collagen deposition. A quantitative measurement for 25 randomly sampled glomeruli was blindly performed on each group using Image J software (1.53 e, U.S. National Institutes of Health, Bethesda, MD, USA).

### 2.3. Detection of Intracellular Superoxide

The ROS generation was assessed by a high-performance liquid chromatography (HPLC) analysis of dihydroethidium (DHE)-derived oxidation products as previously described [55].

### 2.4. NADPH Oxidase Activity

NADPH oxidase activity was measured in the isolated kidney cortex, as previously described [36,37,55,56,57]. Briefly, kidney tissues were homogenized in lysis buffer (20 mM KH_2_PO_4_ [pH 7.0], 1 mM EGTA, 1 mM phenylmethylsulfonyl fluoride, 10 μg/mL aprotinin, and 0.5 μg/mL leupeptin) with 100 strokes in a dounce homogenizer on ice. Total protein concentration was determined using Bio-rad protein assay reagents. To start the assay, 25 μg of homogenates were added to a 50 mM phosphate buffer (pH 7.0) containing 1 mM EGTA, 150 mM sucrose, 5 μM lucigenin, and 100 μM NADPH. Photon emission expressed as relative light units was measured every 30 s for 5 min in a luminometer. A buffer blank (<5% of the cell signal) was subtracted from each reading. Superoxide production was expressed as relative light units (RLU) per minute and per milligrams (mg) of protein.

### 2.5. Inflammatory Markers

Levels of MCP-1, IL-1β, IL-6, IL-17, and TNFα were measured using Elisa kits for each marker (Detroit R & D, Inc., Detroit, MI, USA) according to the manufacturer’s protocol.

### 2.6. 20-HETE Formation

Levels of 20-HETE were measured using the 20-HETE Elisa kit (Detroit R & D, Inc., USA) according to the manufacturer’s protocol.

### 2.7. mRNA Analysis

mRNA was analyzed by real-time RT-PCR using the ΔΔCt method. Total RNA was extracted from the mice kidney cortices using TRIZOL reagent (Sigma-Aldrich, St. Louis, MO, USA) and converted into cDNA using the Revert First Strand cDNA Synthesis Kit (Qiagen) according to the manufacturer’s protocol. cDNA expression was quantified using the CFX96 Touch (Bio-Rad, Hercules, CA, USA) with SYBR Green dye and predesigned mouse RT^2^-quantitative PCR primers. Primers for Fibronectin: Forward 5′-GATGGAATCCGGGAGCTTTT-3′ and Reverse: 5′-TGCAAGGCAACCACACTGAC-3′; Collagen IV: Forward: 5′-GGCGGTACACAGTCAGACCAT-3′ and Reverse: 5′-TGGTGTGCATCACGAAGGA-3′. Primers for YWHAZ: Forward: 5′-GGTGATGACAAGAAAGGAATTGTG-3′ and Reverse: 5′-GCATCTCCTTTTTGCTGATTTCA-3′ or 26s: Forward: 5′-AGGAGAAACAACGGTCGTGCCAAAA-3′ and Reverse: 5′-GCGCAAGCAGGTCTGAATCGTG-3′ was used as an internal reference gene.

### 2.8. Western Blot Analysis

Homogenates from the frozen renal cortex were prepared in 500 µL lysis buffer containing 0.1% sodium dodecyl sulfate (SDS), 0.5% sodium deoxycholate, 150 mM sodium chloride, 50 mM Tris-hydrochloride, 100 mM EDTA, 1% Tergitol (NP40), 100 mM PMSF, 1× of protease inhibitor cocktail containing aprotinin and leupeptin and phosphatase inhibitors cocktail (Bio-world). Homogenates were incubated for 1 h at 4 °C and centrifuged at 10,000× *g* for 30 min at 4 °C. Proteins concentrations in the supernatants were measured using the Lowry quantification method (Bio-rad Laboratory, Hercules, CA, USA). For immunoblotting, proteins (40 μg) were separated on 12.5% polyacrylamide SDS-gel electrophoresis and transferred to nitrocellulose membranes. Blots were incubated with rabbit anti-CYP4A (1:500; Abcam, Cambridge, UK), and Mouse HSC-70 (1:1000; Santa Cruz Biotechnology, Dallas, TX, USA) was used as a loading control. The primary antibodies were detected using horseradish peroxidase-conjugated IgG. Enhanced chemiluminescence helped in visualizing the bands. Densitometric analysis was performed using Image J software (1.53 e, U.S. National Institutes of Health, Bethesda, MD, USA).

### 2.9. Statistical Analysis

Results are expressed as mean ± SD. Statistical significance was assessed by one-way Anova with Prism 9 software (GraphPad Software, San Diego, CA, USA). Significance was determined as a probability (*p*-value) of <0.05.

## 3. Results

### 3.1. Dapagliflozin Treatment Attenuates Functional and Structural Renal Damage in T2DM Mice

First, we confirmed the renoprotective role of dapagliflozin in our animal model. As anticipated, both dapagliflozin and insulin treatments lowered hyperglycemia in the treated diabetic mice groups when compared to the untreated diabetic mice (Table 1). No significant difference was observed in the body weight of the different groups of mice except for the group treated with insulin, which showed reduced body weights when compared to any of the other groups. Furthermore, a significant increase in kidney weight to body weight ratio, which reflects renal hypertrophy, proteinuria (mg/24 h), and elevated urine albumin to creatinine ratio (UACR; µg/mg), was noted in the untreated diabetic mice as compared to control mice. Dapagliflozin as well as insulin treatments markedly reduced the observed kidney failure (Table 1).

**Figure 1:**
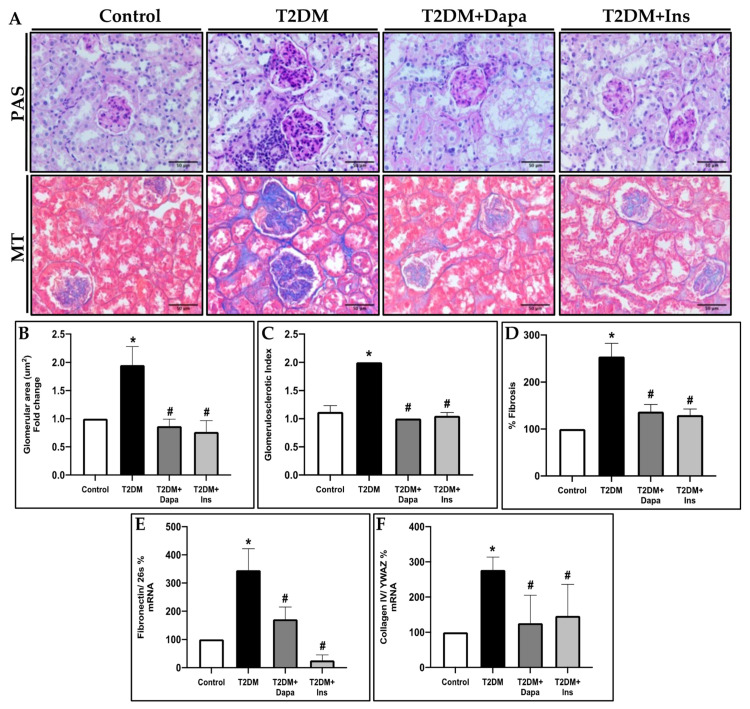
Treatment with dapagliflozin attenuates diabetes–renal fibrosis and ameliorates glomerular injury in T2DM mice. (**A**) Representative images for Masson’s Trichrome (MT) and periodic acid-Schiff (PAS) stains. (**B**) Quantification of the glomerular area and (**C**) glomerulosclerosis index assessed using PAS-stained sections. (**D**) Quantification of the MT-positive percentage reflecting fibrosis. mRNA levels of (**E**) Fibronectin and (**F**) Collagen IV calculated as percentage using the 2^−(ΔΔCt)^ method. Data represent the mean ± SD of 4 mice per group. * *p* < 0.05 relative to the control group; # *p* < 0.05 relative to the untreated diabetic mice group.

These results were paralleled by glomerular and tubular injury in the T2DM mice, in which mesangial expansion, glomerulosclerotic index (GSI), collagen deposition, and fibrosis were all increased when compared to their non-diabetic counterparts. Treatment with dapagliflozin or with insulin showed a significant reduction in these same histopathological parameters (Figure 1A–D). Consistent with the GSI and mesangial expansion findings, glomerular collagen deposition was increased in the untreated diabetic mice (Figure 1A,D). Renal injury was further validated by measuring the gene expression of classical extracellular matrix (ECM) molecules. Our results demonstrate an evident increase in the renal expression of Fibronectin (Figure 1E) and Collagen IV (Figure 1F) in the untreated T2DM group. The treatment with dapagliflozin and insulin significantly reversed the observed increase in the fibrotic markers (Figure 1E,F).

### 3.2. Dapagliflozin Inhibits CYP4A-Induced 20-HETE Production and Attenuates Oxidative Stress in the Kidneys of T2DM Mice

**Figure 2:**
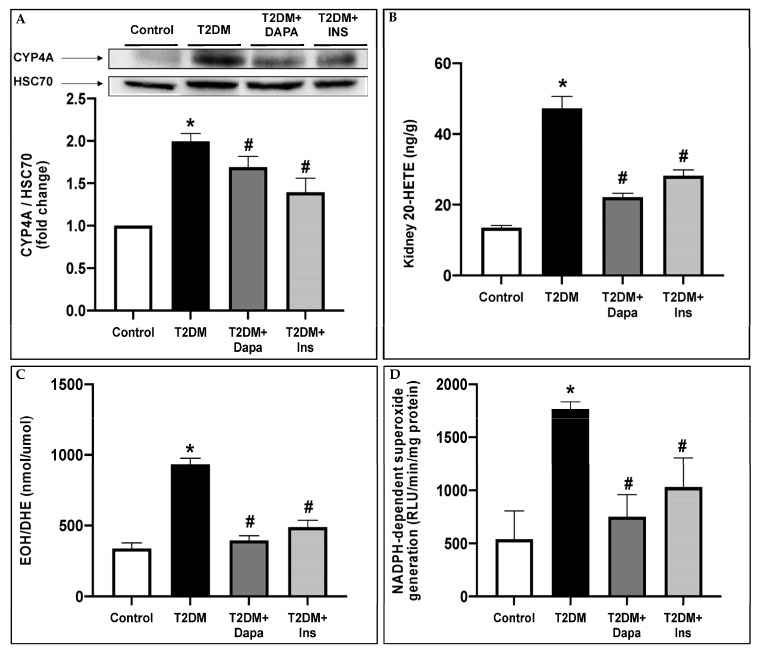
Dapagliflozin prevents the overproduction of 20-HETE, downregulates CYP4A protein expression, and attenuates ROS generation and NADPH oxidase activity in type 2 diabetic mice. (**A**) Representative Western blots showing the expression of CYP4A and CYP4A/HSC70 quantification presented as fold change. (**B**) Histograms representing 20-HETE formation measured in the renal cortex of the different groups of mice. (**C**) HPLC analysis and quantification of EOH/DHE ratios (nmol/umol), and (**D**) NADPH-dependent superoxide generation (RLU/min/mg protein) measured in the renal cortex of the different groups of mice. Data represent the mean ± SD of 4 mice per group. * *p* < 0.05 relative to the control group; # *p* < 0.05 relative to the untreated diabetic mice group.

We have previously demonstrated that CYP4A-induced 20-HETE production is implicated in the pathogenesis of DKD by inducing ROS production and NADPH oxidase activity [35,36,37]. In this study, we demonstrate that dapagliflozin mitigates the diabetes-increased CYP4A protein expression and 20-HETE production, which coincided with a decrease in ROS production and NADPH oxidase activity (Figure 2A–D).

### 3.3. Treatment with Dapagliflozin Reduces the Systemic and Renal Inflammation Observed in the T2DM Mice

**Figure 3:**
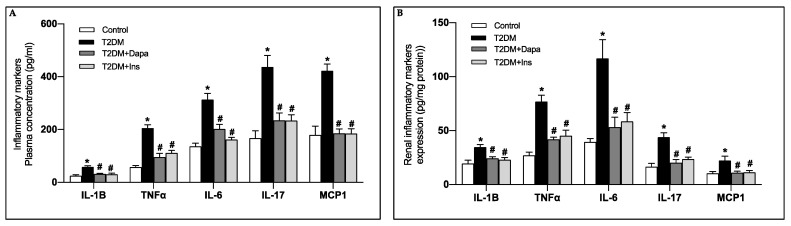
Dapagliflozin ameliorates diabetes-induced renal inflammation as assessed by the reduction of renal inflammatory markers. MCP-1, IL-1β, IL-6, IL-17 and TNFα levels assessed in the (**A**) circulation and the (**B**) renal cortex of the different groups of mice. Data represent the mean ± SD of 4 mice per group. * *p* < 0.05 relative to the control group; # *p* < 0.05 relative to the untreated diabetic mice group.

Inflammation has been described to play a role in the development of DKD through the increased cytokine production and exacerbation of oxidative stress [39]. The expression profiles of the essential proinflammatory cytokines and chemokines, MCP-1, IL-1β, IL-6, IL-17, and TNFα, were significantly increased in plasma and kidney cortices of the untreated diabetic mice when compared to their control littermates (Figure 3A,B). Interestingly, the observed increase in the inflammatory mediators was attenuated when the diabetic mice were treated with dapagliflozin or insulin (Figure 3A,B).

### 3.4. CYP4A/20-HETE Inhibition by HET0016 Attenuates Renal Injury in T2DM

**Figure 4:**
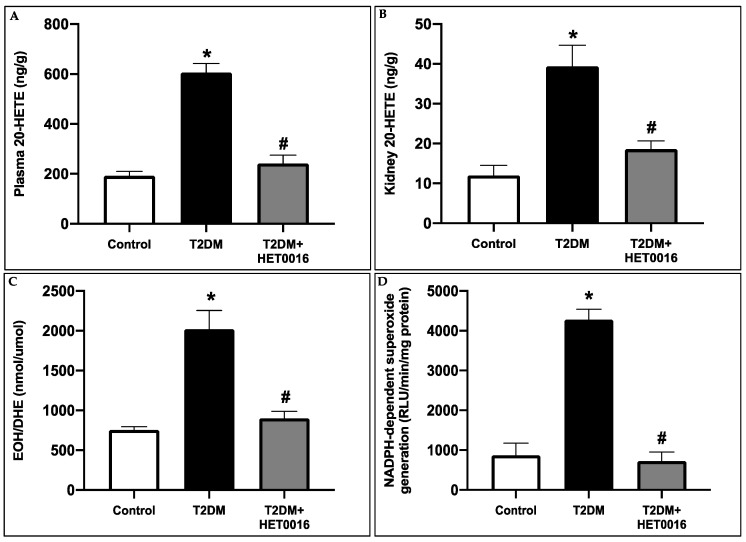
CYP4A-dependant 20-HETE production contributes to diabetes-induced ROS overproduction and NADPH oxidase activity in the kidney cortices of T2DM mice. 20-HETE levels measured in (**A**) the plasma and (**B**) the renal cortices of the different groups of mice. (**C**) HPLC analysis and quantification of EOH/DHE ratios (nmol/umol), and (**D**) NADPH-dependent superoxide generation (RLU/min/mg protein) measured in the renal cortex of the different groups of mice. Data represent the mean ± SD of 5 mice per group. * *p* < 0.05 relative to the control group; # *p* < 0.05 relative to the untreated diabetic mice group.

In these set of experiments, we used control and type 2 diabetic FVB/NJ mice to assess whether the observed diabetes-induced renal changes are reproducible across strains. Furthermore, these experiments will allow us to determine whether the inhibition of 20-HETE plays a central role in the pathogenesis of diabetes-induced renal damage. By utilizing multiple strains of mice and examining the impact of 20-HETE inhibition on renal function and structure, this study will allow us to gain a more comprehensive understanding of the mechanisms underlying diabetic kidney disease. As expected, the 20-HETE production was significantly higher in the plasma and kidneys of untreated type 2 diabetic mice as compared to the controls. This was prevented or reduced in the diabetic animals treated with HET0016, a potent inhibitor of CYP4A and therefore of 20-HETE production (Figure 4A,B). The elevated 20-HETE levels correlated with an overproduction of ROS as determined by HPLC, which was paralleled by an increase in NADPH oxidase enzymatic activity in the untreated diabetic mice compared to control mice. This increase was inhibited in the diabetic mice after HET0016 treatment (Figure 4C,D).

Moreover, our results demonstrate that HET0016 decreases renal hypertrophy as assessed by kidney weight to body weight ratio and restores the levels of UACR and proteinuria to near control levels. Of interest, HET0016 did not affect the glycemia of the treated T2DM mice, suggesting that 20-HETE production plays a central role in diabetes-induced renal injury, and its inhibition is renoprotective independently of hyperglycemia (Table 2).

**Figure 5:**
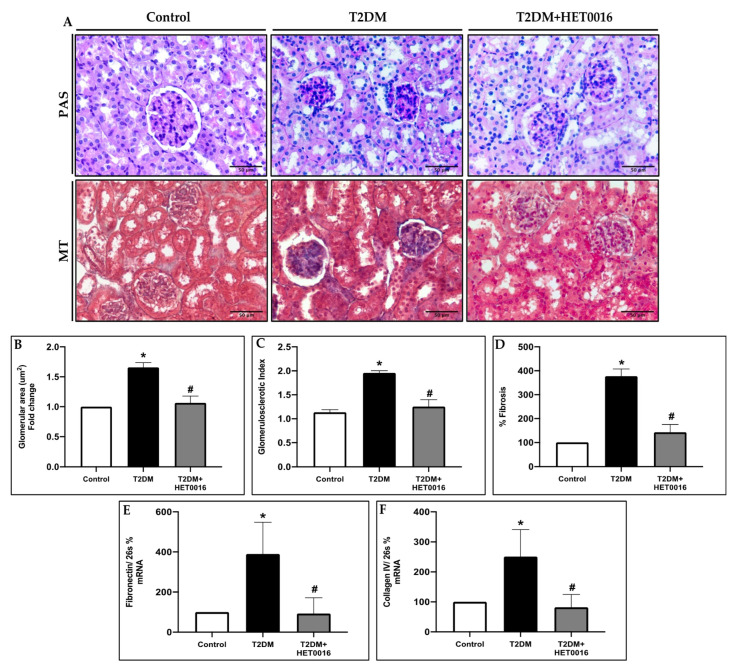
20-HETE production mediates diabetes-induced renal fibrosis and glomerular injury in T2DM mice. (**A**) Representative images for Masson’s Trichrome (MT) and periodic acid-Schiff (PAS) stains. (**B**) Quantification of the glomerular area and (**C**) glomerulosclerosis index assessed using PAS-stained sections. (**D**) Quantification of the MT-positive percentage reflecting fibrosis. mRNA levels of (**E**) Fibronectin and (**F**) Collagen IV calculated as percentage using the 2^−(ΔΔCt)^ method. Data represent the mean ± SD of 5 mice per group. * *p* < 0.05 relative to the control group; # *p* < 0.05 relative to the untreated diabetic mice group.

In parallel, the histopathological assessment of renal tissues of the different groups of mice shows that HET0016 treatment attenuates diabetes-induced glomerular and tubular injury as assessed by the decrease in glomerular hypertrophy (Figure 5A,B) glomerulosclorosis (Figure 5A,C), and mesangial expansion (Figure 5A,D). These results were paralleled by a decrease in tubulointerstitial fibrosis and collagen IV deposition in the diabetic mice after HET0016 treatment (Figure 5E,F).

### 3.5. CYP4A-Induced 20-HETE Production Prompts the Increase in Reactive Oxygen Species Production and the Rise in Proinflamatory Markers

**Figure 6:**
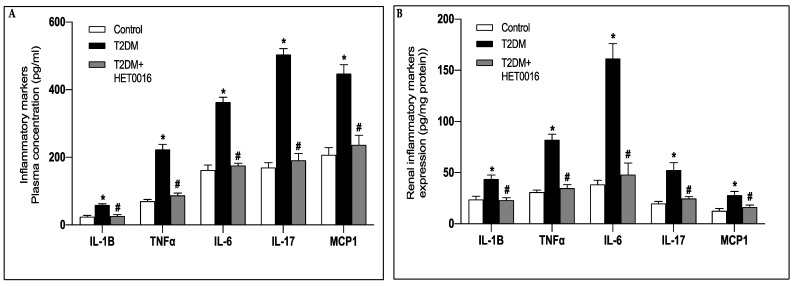
Inhibition of CYP4A-dependant 20-HETE production mitigates renal inflammation in T2DM mice as assessed by the reduction of renal inflammatory markers. MCP-1, IL-1β, IL-6, IL-17 and TNFα levels assessed in the (**A**) circulation and the (**B**) renal cortex of the different groups of mice. Data represent the mean ± SD of 5 mice per group. * *p* < 0.05 relative to the control group; # *p* < 0.05 relative to the untreated diabetic mice group.

The anti-inflammatory effect of inhibiting CYP4A/20-HETE in DKD was assessed by measuring MCP-1, IL-1β, IL-6, IL-17, and TNF⍺ in the plasma (Figure 6A) and the kidney cortices (Figure 6B) of the different groups of mice. Our results demonstrate that the inhibition of 20-HETE production by HET0016 results in a significant reduction in the measured proinflammatory markers observed in the T2DM mice (Figure 6A,B).

Collectively, our findings strongly support the notion that the activation of CYP4A/20-HETE plays a pivotal role in the pathogenesis of diabetic kidney disease (DKD), and that the renoprotective effects of dapagliflozin, an SGLT2 inhibitor, are mediated via the inhibition of CYP4A/20-HETE. This inhibition effectively reduces both systemic and renal inflammation, as well as reactive oxygen species (ROS) production, thus highlighting a novel therapeutic target for DKD.

## 4. Discussion

SGLT2i are a class of antihyperglycemic medication proposed to play a renoprotective role in patients with diabetes through both glucose-lowering dependent and independent mechanisms [12,13,14,15,16]. The American Diabetes Association (ADA) recommends using an SGLT2i for patients with T2DM and DKD when eGFR ≥ 20 mL/min/1.73 m^2^ since its glucose-lowering efficacy is directly proportional to the glomerular filtration. However, the post hoc analysis of Canagliflozin and Renal Events in Diabetes with Established Nephropathy Clinical Evaluation (CREDENCE) trial suggests that the SGLT2i canagliflozin reduces the progression of kidney disease even in patients with low eGFR [58]. Moreover, results from the DAPA-HF (Dapagliflozin and Prevention of Adverse Outcomes in Heart Failure) trial demonstrate that dapagliflozin improves cardiovascular outcomes regardless of the presence or absence of diabetes [59]. Thus, a growing series of observations suggest that the renal and cardiovascular benefits of SGLT2i are also mediated through glucose-independent mechanisms.

In the current study, we intended to investigate the mechanism behind the renoprotective effects of the SGLT2i in the context of diabetes. To our knowledge, this is the first study to suggest that the downregulation of CYP4A/20-HETE contributes at least partially to the nephroprotective actions exerted by SGLT2i. We used a HFD/STZ-induced T2DM mouse model for our experiment. The combination of HFD followed by the low dose STZ treatment can mimic the natural history and metabolic characteristics of T2DM in humans, including impaired glucose tolerance, obesity, insulin resistance, and hyperglycemia [60,61]. Consistent with the earlier observations from the literature, SGLT2 inhibition by dapagliflozin prevents some of the major hallmarks of renal dysfunction and DKD, including hypertrophy, as revealed by the increased kidney weight to body weight ratio, proteinuria, and elevated urinary ACR in our diabetic mice. Moreover, DKD is accompanied by phenotypic changes at the level of kidney glomeruli, such as mesangial expansion and ECM accumulation leading to fibrosis [62]. As anticipated, inhibiting SGLT2 in our T2DM mouse model protected the renal tissue from these histopathological changes as shown by the decreased GSI, glomerular area, and expression levels of the markers of fibrosis, fibronectin, and collagen IV. However, whether the renoprotective effects of SGLT2i are solely due to glucose control or are related to glucose-independent pathways remains to be determined.

To further delineate the contribution of the CYP4A/20-HETE axis to our proposed mechanism of renoprotection in T2DM, the HFD/STZ-induced diabetic mice were treated with HET0016. HET0016 is a potent and selective inhibitor of the CYP enzymes which catalyze the synthesis of 20-HETE from AA [63]. Growing evidence has implicated the role of 20-HETE, an endogenous CYP4A/F metabolite of AA, in vascular and kidney injury. Nevertheless, depending on its site of production, different levels of 20-HETE can have various and even opposing functions [35,36]. Previously published works by our group demonstrated that HET0016 decreases CYP4A protein expression [36,64] and thus CYP4A protein levels were not measured in animals treated with HET0016. Thus, measuring the decrease in 20-HETE was sufficient to confirm the effectiveness of the treatment. Albeit the central role of 20-HETE in the regulation of renal function, its impact on DKD needs further clarification. Gangadhariah et al. suggested that 20-HETE exacerbates renal injury in STZ-induced diabetic mice [65]. In addition, in a kidney ischemia/reperfusion injury rat model, the inhibition of CYP4A/F by HETE0016 or treatment with a 20-HETE antagonist (6,15,20-HED) offered a renoprotective effect [66,67]. Furthermore, in an STZ-induced diabetic rat model, the increased production of 20-HETE was associated with the overexpression of fibronectin and transforming growth factor-β1 (TGF-β1) in the kidneys of the experimental rodents [68], where these molecules are proven to be a profibrotic factor in DKD [69]. Our group has previously mirrored these conclusions, with prior studies demonstrating that hyperglycemia/diabetes induces renal CYP4A expression and consequently increases 20-HETE production; these changes are proposed to be implicated in the pathogenesis of DKD [35,36,37]. On the other hand, Luo et al. demonstrated that in diabetic rats, DKD can induce excessive production of TGF-β1 in the glomerulus, paralleled with a reduction in 20-HETE levels [70].

It is established that CYP450 is a significant source of cellular ROS in different tissues, including the renal tissue [30,31,32,37]. On the other hand, CYP450 eicosanoids have wide range of biological effects, including vascular tone regulation, cellular proliferation, renal tubular transport, and inflammation [28]. Former clinical studies have established an association between urinary 20-HETE excretion, oxidative stress, and endothelial dysfunction in human subjects [32,71]. Another recent finding indicated that upregulated endothelial CYP4A-derived 20-HETE contributes to enhanced superoxide production and vascular oxidative stress in an insulin-resistant obese rat model [72]. We have also reported earlier that diabetes-induced oxidative stress was associated with CYP4A upregulation and 20-HETE overproduction in the kidneys of a type 1 diabetes animal model [35,37]. In fact, 20-HETE is a well-known proinflammatory mediator and its overexpression induces NF-κB activation and cytokine expression in endothelial cells. Ishizuka et al. demonstrated that the treatment of endothelial cells with 20-HETE triggers the NF-κB activation and ROS production leading to elevated IL-8 levels and intracellular adhesion molecules, as well as subsequent endothelial cell dysfunction [73]. Together, these studies establish 20-HETE as a key mediator of vascular and renal inflammation and oxidative stress.

Although ROS play an important role in cell signaling, their overproduction in the kidneys under pathological conditions including diabetes is associated with renal inflammation. Diabetes-induced ROS production stimulates the recruitment of numerous inflammatory cells, where the infiltration of macrophages and T cells plays a crucial role in initiating renal damage in DKD [74]. Immune cell recruitment and activity are usually modulated by MCP-1 [75]. Of importance, MCP-1 is found to be predominantly expressed in renal monocytes, endothelial cells, tubular epithelial cells, and mesangial cells [43,76,77,78,79,80] and is highly regulated by proinflammatory cytokines, namely TNFα and IL-1 [81]. Upregulation of MCP-1 levels was described in patients with DKD [43], and elevations were also noted in the glomeruli [82] and tubulointerstitium [83] of experimental models of type 1 diabetes. Interestingly, NF-κB was the main transcriptional factor reported to be implicated in initiating the inflammatory response in diabetes [84]. NF-κB activation induces the expression of proinflammatory genes, including MCP-1, IL-6, and TNFα [85,86,87,88] which are all key contributors to the development of DKD. In fact, TNFα and IL-6 levels were demonstrated to be strongly linked to renal disease progression. Several studies demonstrated the significant role of TNFα in ROS production [87,89], while IL-6 has been demonstrated to promote mesangial cell proliferation, ECM accumulation, and enhance endothelial cell permeability [88,90]. Another relevant cytokine found to be involved in renal inflammation is IL-17. Interestingly, IL-17 has been demonstrated to induce the expression of MCP-1, IL-1β, IL-6, and TNFα in tubular and mesangial cells leading to local macrophage recruitment [91,92]. A recent study has established the upregulation of TNFα, IL-6, and IL-1β upon treating podocytes or tubular epithelial cells with recombinant IL-17 under hyperglycemic conditions in vitro [53]. These observations were supported by their in vivo findings, where the IL-17^−/−^ diabetic mice demonstrated a reduction in albuminuria, renal fibrosis, and glomerular injury [53]. In congruence with the aforementioned observations, we demonstrate that in our experimental model of T2DM, inhibiting CYP4A-derived 20-HETE by HET0016 reverses the diabetes-associated NADPH-dependent superoxide generation and ROS overproduction. Consequently, the HET0016 treatment prevents renal inflammation in diabetic mice, as suggested by the decreased circulatory and renal levels of the proinflammatory markers measured. Thus, while we acknowledge that investigating the combined effect of HET0016 and dapagliflozin is important and promising for future studies, this goes beyond the scope of our manuscript aiming at elucidating the individual mechanisms of the action of dapagliflozin as a renoprotective agent.

**Figure 7:**
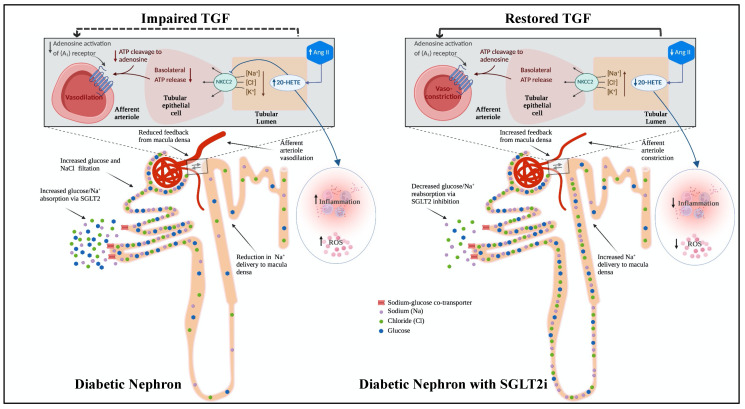
Proposed renoprotective mechanism of sodium/glucose co-transporter 2 (SGLT2) inhibition on diabetic nephron. In diabetic kidney, the overexpression and increased activity of SGLT2 on glucose and sodium reabsorption in the proximal convoluted tubule leads to decreased delivery of solutes to the macula densa. This aberrant transport into the tubular epithelial cells results in reduction in the adenosine triphosphate (ATP) release from the basolateral membrane and limits its conversion to adenosine. On the other hand, increased 20-HETE renal synthesis associated with diabetes and increased Angiotensin II (Ang II) production also inhibits the apical Na^+^-K^+^-2Cl^−^ (NKCC2) cotransporter, which is responsible for NaCl uptake in the macula densa cells. Consequently, reduced activation of A1 adenosine receptor expressed in the afferent arteriole leads to vasodilation and impaired tubuloglomerular feedback (TGF). After treatment with SGLT2 inhibitor, the solute delivery to the macula densa is increased in the diabetic nephron, and decreased Ang II and 20-HETE production reverses the NKCC2 inhibition. Therefore, the basolateral release of ATP from the tubular epithelium is increased. This in turn will restore adenosine activation of the A1 receptor resulting in vasoconstriction of the afferent arteriole. At the same time, 20-HETE inhibition attenuates reactive oxygen species (ROS) production and inflammation associated with diabetic kidney disease.

SGLT2i have been demonstrated to be involved in reversing molecular processes related to inflammation, fibrosis, and ECM turnover [93]. In animal models of DKD, SGLT2i have been reported to decrease markers of inflammation and oxidative stress [94]. Indeed, our results are consistent with the former findings, demonstrating that inhibiting SGLT2 in the T2DM animal model averted the increase in inflammatory mediators and ROS production, eventually preventing diabetes-induced renal injury. Notably, our data demonstrate that treatment with SGLT2i attenuated the increase in the renal CYP4A expression and 20-HETE production observed in T2DM mice. This could be explained by the changes in glomerular function evoked during tubuloglomerular feedback (TGF) and the angiotensin II (AngII) actions. AngII plays a key role in modulating the vascular tone of the afferent arteriole [95]. Reduction in the delivery of sodium chloride (NaCl) to the macula densa cells of the juxtaglomerular apparatus (JGA) leads to an increased GFR and intraglomerular pressure through TGF [96]. In diabetes, hyperglycemia leads to increased sodium-coupled glucose reabsorption by the proximal tubules and decreased sodium delivery to the macula densa [94]. Consequently, the conversion of ATP into adenosine is inhibited, which reduces the levels of this potent vasoconstrictor leading to vasodilation of the afferent arteriole and causing increased renal plasma flow (RPF), intraglomerular pressure, and eventually hyperfiltration [94]. SGLT2i will enhance the sodium delivery to the macula densa, thus generating signals that provoke the afferent arteriole vasoconstriction, reducing RPF, improving the intraglomerular pressure, and ultimately curbing the progression of DKD [94]. On the other hand, 20-HETE is also a key modulator in the TGF response in the kidneys. In the thick ascending limb of Henle (TALH), 20-HETE has been demonstrated to inhibit the apical Na^+^-K^+^-2Cl^−^ (NKCC2) cotransporter [97,98]. Moreover, it is essential for TGF response, as the major uptake mechanism of NaCl in the macula densa cells of TALH is mediated by NKCC2 [99,100], and the inhibition of this critical transporter can lead to a complete blockade of the TGF response [101]. Wang et al. demonstrate a marked effect exerted by NKCC inhibitors on reducing the reactivity of the afferent arteriole, the principal effector limb of TGF, to elevated pressure and AngII, which heavily affects the arteriolar vascular tone [99]. More importantly, several studies have previously reported that augmented AngII levels increase the renal synthesis of 20-HETE [102,103,104,105]. Another significant association was made between intrarenal RAAS and SGLT2 expression in humans and mice. Treatment with SGLT2i has been demonstrated to attenuate the AngII-induced hypertensive renal injury in mice [106]. Similarly, the urinary AngII levels were significantly decreased in the T2DM rat model when treated with dapagliflozin [107]. Thus, the influence of SGLT2i on 20-HETE could be mediated by AngII. In short, SGLT2i could exert their renoprotective effects in a two-way mechanism mediated in both cases by a RAAS-dependent decrease in the levels of 20-HETE (Figure 7). On one hand, decreased levels of 20-HETE enhance the sodium transport in the TALH, hence improving TGF. On the other hand, low levels of 20-HETE attenuate oxidative stress and renal inflammation induced by the diabetic milieu.

Moreover, several randomized controlled trials have suggested that SGLT2 inhibitors can decrease the daily dose of insulin [108], which can be explained, to a certain extent, by the lower 20-HETE levels caused by SGLT2 inhibition, allowing a better utilization of the administered insulin. This mechanism is likely related to the observed reduction in oxidative stress and inflammation, as well as the amelioration of renal fibrosis and sclerosis, which were demonstrated in our study. The potential interaction between dapagliflozin and insulin warrants further investigation to fully elucidate the mechanism of action of SGLT2 inhibitors in diabetic kidney disease.

Although our findings were based on animal models, further investigations using cultured cells could help validate our results. We acknowledge that this could be considered a minor limitation of our study. Of importance, our study adds to previous research, by our group and others [35,36], that have also demonstrated the renoprotective effects of inhibiting the 20-HETE production in cultured glomerular and renal tubular cells. Moreover, while other studies have suggested the potential benefits of dapagliflozin in cultured tubular cells [35,36], the precise mechanisms involved are still being investigated by several research groups. Our findings, however, provide new insights into the role of dapagliflozin in DKD, which could serve as a cornerstone in future research.

## 5. Conclusions

In conclusion, to our knowledge this is the first study to investigate the role of dapagliflozin on cytochrome P450 of the 4A family (CYP4A) and its metabolite 20-HETE. Hereby, it is demonstrated that T2DM induces the activation of CYP4A and subsequent 20-HETE overproduction, leading to an increased production of ROS and inflammation. Targeting this crosstalk by SGLT2i represents a specific and promising therapeutic strategy in the management of T2DM-induced DKD.

## Figures and Tables

**Table 1:** Glucose level, body weight, kidney weight to body weight ratio, proteinuria and urinary albumin to creatinine ratio of control mice, untreated T2DM mice, and T2DM mice treated with dapagliflozin or insulin. Data represent the mean ± SD of 4 mice per group. * *p* < 0.05 relative to the control group; ^#^ *p* < 0.05 relative to the untreated diabetic mice group.

	**Control**	**T2DM**	**T2DM + Dapa**	**T2DM + Ins**
Glucose levels (mg/dL)	143 ± 17	302 ± 55 *	214 ± 16 ^#^	196 ± 30 ^#^
Body weight (g)	32.93 ± 2.91	35.6 ± 1.06	33.15 ± 3.55	30.55 ± 2 ^#^
Kidney Weight/ Body weight (mg/g)	6.6 ± 0.33	8.5 ± 0.19 *	6.9 ± 0.37 ^#^	6.5 ± 0.35 ^#^
Proteinuria (mg/24 h)	26 ± 1.71	68 ± 17.57 *	28 ± 5.59 ^#^	21 ± 3.47 ^#^
UACR (μg/mg)	31 ± 5	88 ± 14 *	13 ± 4 ^#^	21 ± 6 ^#^

**Table 2:** Glucose level, body weight, kidney weight to body weight ratio, proteinuria and urinary albumin to creatinine ratio of control mice, untreated T2DM mice, and T2DM mice treated with HET0016. Data represent the mean ± SD of 5 mice per group. * *p* < 0.05 relative to the control group; ^#^ *p* < 0.05 relative to the untreated diabetic mice group.

	**Control**	**T2DM**	**T2DM + HET0016**
Glucose levels (mg/dL)	154 ± 19	485 ± 74 *	476 ± 85 *
Body weight (g)	32 ± 0.51	34 ± 2.62	37 ± 1.96 *
Kidney Weight/ Body weight (mg/g)	7.3 ± 0.27	9 ± 0.45 *	7.5 ± 0.3 ^#^
Proteinuria (mg/24 h)	24 ± 6.76	112 ± 63.26 *	25 ± 11.63 ^#^
UACR (ug/mg)	52 ± 6	198 ± 10 *	70 ± 5 ^#^

## Data Availability

The data presented in this study are available on request from the corresponding author upon reasonable request.
